# Correction to “Comprehensive analysis of circular RNA profiling in AZD9291‐resistant non‐small cell lung cancer cell lines”

**DOI:** 10.1111/1759-7714.15392

**Published:** 2024-07-31

Chen T, Luo J, Gu Y, Huang J, Luo Q, Yang Y. Comprehensive analysis of circular RNA profiling in AZD9291‐resistant non‐small cell lung cancer cell lines. *Thorac Cancer*. 2019;10(4):930–41. https://doi.org/10.1111/1759-7714.13032.

In paragraph 8, subheading with Microarray data analysis, the text “The required microarray information has been imputed into the Gene Expression Omnibus (serial number: GSE48885).” was incorrect.

This should have read: “The required microarray information has been imputed into the Gene Expression Omnibus (serial number: GSE263003).”

We apologize for this error.

